# Supplementary material for: Investigating microbial population structure and function in the chicken caeca and large intestine over time using metagenomics
Source: BMC Res Notes. 2025 Aug 15;18:355. doi: 10.1186/s13104-025-07441-7 (PMC12357378; doi:10.1186/s13104-025-07441-7)
Supplement: Supplementary file 2 — Recovered nutrient cycles including Carbon, Nitrogen, and Sulphur cycles for all the metagenomic assembled genomes given as PDF images, and identifiable through bin numbers. [file 13104_2025_7441_MOESM2_ESM.zip › Supplementary Data2/bin.22.draw_other_cycle_single.pdf]

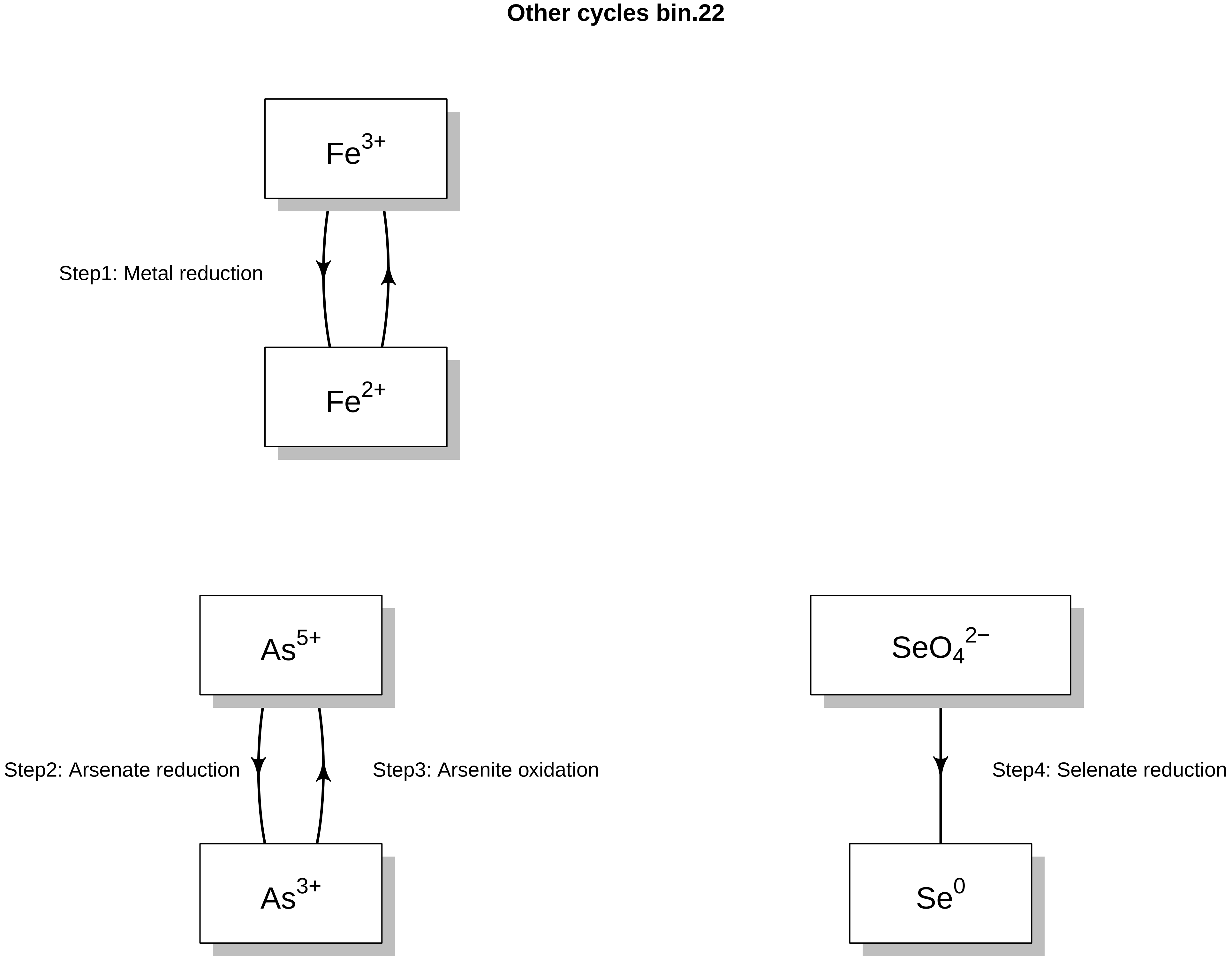
Other cycles bin.22
Fe^{3+}
Step1: Metal reduction
Fe^{2+}
As^{5+}
Step2: Arsenate reduction
Step3: Arsenite oxidation
As^{3+}
SeO_4^{2-}
Step4: Selenate reduction
Se^0
